# Patient Perceptions of e-Visits: Qualitative Study of Older Adults to Inform Health System Implementation

**DOI:** 10.2196/45641

**Published:** 2023-05-26

**Authors:** Timothy J Judson, Meera Subash, James D Harrison, Jan Yeager, Aimée M Williams, Carrie K Grouse, Maria Byron

**Affiliations:** 1 Department of Medicine University of California San Francisco San Francisco, CA United States; 2 Houston School of Biomedical Informatics UTHealth Houston, TX United States; 3 Clinical Innovation Center University of California San Francisco San Francisco, CA United States; 4 Department of Neurology University of California San Francisco San Francisco, CA United States

**Keywords:** e-visit, patient portal message, digital health tool, patient portal, perception, attitude, qualitative, e-consult, remote care, remote visit, remote consult, vulnerable, messaging, telehealth, telemedicine, eHealth

## Abstract

**Background:**

Electronic visits (e-visits) are billable, asynchronous patient-initiated messages that require at least five minutes of medical decision-making by a provider. Unequal use of patient portal tools like e-visits by certain patient populations may worsen health disparities. To date, no study has attempted to qualitatively assess perceptions of e-visits in older adults.

**Objective:**

In this qualitative study, we aimed to understand patient perceptions of e-visits, including their perceived utility, barriers to use, and care implications, with a focus on vulnerable patient groups.

**Methods:**

We conducted a qualitative study using in-depth structured individual interviews with patients from diverse backgrounds to assess their knowledge and perceptions surrounding e-visits as compared with unbilled portal messages and other visit types. We used content analysis to analyze interview data.

**Results:**

We conducted 20 interviews, all in adults older than 65 years. We identified 4 overarching coding categories or themes. First, participants were generally accepting of the concept of e-visits and willing to try them. Second, nearly two-thirds of the participants voiced a preference for synchronous communication. Third, participants had specific concerns about the name “e-visit” and when to choose this type of visit in the patient portal. Fourth, some participants indicated discomfort using or accessing technology for e-visits. Financial barriers to the use of e-visits was not a common theme.

**Conclusions:**

Our findings suggest that older adults are generally accepting of the concept of e-visits, but uptake may be limited due to their preference for synchronous communication. We identified several opportunities to improve e-visit implementation.

## Introduction

Electronic visits (e-visits) allow patients to get web-based medical advice without the need for a face-to-face visit. They are billable, asynchronous patient-initiated messages that are sent through a patient portal and require at least five minutes of medical decision-making by a provider [1]. There are several benefits of e-visits to patients. They provide a flexible option for obtaining medical care that does not require travel or time off from work, which may save patients time and money [2,3]. Studies have demonstrated improvements in access to care for patients in rural areas [4-6] and equivalent patient outcomes at a lower cost [7]. For physicians, e-visits are attractive because unlike other patient portal messages, they provide a mechanism for reimbursement [8], and therefore, may lead to direct compensation or credit toward productivity targets.

e-Visits have been used for the management of chronic conditions and for consultation on nonurgent acute health concerns [9,10]. The use and flexibility for reimbursement of e-visits has increased in recent years [11,12]. In 2020, the Centers for Medicare and Medicaid services began to cover e-visits in all types of locations, including the patient’s home, and in all areas of the country, rather than just rural areas [12]. Particularly during the COVID-19 pandemic, e-visits have become an increasingly important means of providing virtual care [12].

e-Visits are only available to patients who use patient portals, creating the potential for health disparities. Specifically, older adults and those from minority backgrounds are less likely to enroll in patient portals [13-15]. Barriers to using digital tools like patient portals include limited internet access, low computer skills, and strong habits associated with face-to-face or phone scheduling [16]. As health systems shift toward providing more virtual care [17,18], it is crucial that we understand the interest and ability of vulnerable patient groups to use these digital tools so as to prevent worsening health disparities. Furthermore, when health systems begin to offer e-visits, they may see low uptake and dissatisfaction if key patient groups are uncomfortable with their use. Although several studies have evaluated the demographic characteristics of patients who use e-visits [9,10,19] and providers who offer them [4,7,20], little is known about how patients at risk of digital health disparities perceive this visit type, particularly compared to traditional, nonbillable patient portal messages. Therefore, in this qualitative study, we aimed to understand patient perceptions of e-visits, including their perceived utility, barriers to use, and care implications, with a focus on vulnerable patient groups.

## Methods

### Study Setting

We conducted this study at University of California, San Francisco (UCSF) Health, a large tertiary academic medical center that originally introduced e-visits in 2020 and was considering a change to how e-visits were offered to patients. UCSF uses a commercially available electronic health record from Epic Systems. Over 90% of patients empaneled to UCSF primary care were enrolled in the patient portal during the time of the study.

### Study Design and Oversight

We conducted a qualitative study using in-depth structured individual interviews with patients from diverse backgrounds to assess their knowledge and perceptions surrounding e-visits as compared with portal messages and other visit types. We specifically explored their perceptions of the acceptability and usability of e-visits.

### Ethical Considerations

This study was reviewed and deemed exempt by the University of California at San Francisco Institutional Review Board.

### Study Recruitment

Potential participants were identified from among patients who received primary care at UCSF Health. To include patients with varying degrees of comfort using web-based patient portals for their care, we recruited half of the participants from among patients who had used a patient portal–based triage tool [21] and half of the participants from among patients who had used an identical telephone-based triage tool in the past 6 months. We hypothesized that patients who had opted to use the patient portal tool were more likely to be comfortable using other patient portal tools and that patients who had opted to use the more time-consuming telephone tool might be less comfortable or less preferential toward using other patient portal tools, such as e-visits.

To ensure representation from a diverse sample of patients, we then further identified those who met the following criteria: Latinx ethnicity, African American race, having MediCal (Medicaid) insurance, living outside of the 9 San Francisco Bay Area counties, and non–English speaking. We randomly selected at least two participants from each of these groups before recruiting a random selection of patients, regardless of their demographic characteristics, until we reached saturation of responses. Race and ethnicity were treated as social rather than biologic constructs and were included as a proxy for unmeasured factors experienced by socially marginalized populations that may predict their experience using e-visits. We used MediCal insurance as a proxy for low socioeconomic status. The researchers did not have previous treatment relationships with the participants.

On the initial phone call, the research coordinator described the study, and for those interested in participating, obtained informed consent and scheduled an interview. Participants were given a US $60 gift card for their participation, which was sent to them prior to completing the interview.

### Participant Interviews

A trained service designer (JY) with extensive experience in human-centered design and qualitative research conducted an approximately 60-minute interview with each participant. Interviews were conducted by Zoom videoconferencing, if possible. If the participant was unable to use video, they were conducted by telephone. If patients preferred, they could invite a caretaker, such as an adult child, to join the interview. The service designer used a structured interview guide (Multimedia Appendix 1), which included the following domains: current methods of communicating with providers, perceptions of e-visits and patient portal messages, financial aspects of e-visits, naming conventions for e-visits, self-assessment of health technology literacy, and current health status. This interview guide, consisting of mostly open-ended questions, was developed after conducting a review of literature and lay press to identify potential risks and benefits of e-visits, particularly compared to other visit types. Certain questions were adapted from validated questionnaires [22,23]. Participants could elect not to answer any question. Patient interviews were digitally recorded and transcribed using the transcription service Tigerfish [24].

### Transcription Review and Analysis

We used a combination of qualitative and quantitative content analysis to analyze interview data [25,26]. Transcripts were reviewed independently by 2 clinician investigators (TJJ and MS) under the guidance of a trained qualitative researcher (JDH). We organized data analysis around the study questions: acceptability, usability, as well as financial and care implications. We first used qualitative content analysis to systematically examine the transcripts to obtain a condensed understanding and description of content [25]. We used a data-driven (inductive) approach to analysis whereby open coding was performed to identify salient and elevated topics of importance within the data set. To ensure trustworthiness, throughout analysis, reviewers (TJJ, MS, and JDH) met to refine and define coding categories, and coding disparities were discussed and resolved by negotiated consensus [27]. Coding categories were then grouped into higher-order categories or themes.

Quantitative content analysis was then performed to count coding categories. This was conducted for the purpose of providing a more detailed assessment of how frequently certain themes or codes were mentioned. For each code, there was at least one corresponding question in the interview guide pertaining to that topic. For the purposes of determining the proportion of responses related to a particular code or coding category, we excluded participants from the denominator if they did not answer the pertinent question(s). We then did a secondary analysis comparing themes between patients who had used the patient portal tool in the past 6 months, compared to those who had not. We aimed to enroll until we reached saturation of responses, which we defined as having done at least 9-17 interviews [28] and observing the repetition of themes without significant new insights.

### Sharing Findings With the Health System

After analyzing qualitative themes and identifying key patient responses, we shared findings with health systems leadership to inform informatics and operational changes related to the broader implementation of e-visits at the institution.

## Results

From April 2021 to June 2021, we conducted qualitative interviews with a total of 20 adults (Table 1). The median age was 74 (IQR 68.5-77.8) years. Of 20 participants, 13 (65%) identified as male, and 7 (35%) identified as female; 10/20 (50%) participants identified as White or Caucasian, 5/20 (25%) as Asian, 2/20 (10%) as Black or African American, 1/20 (5%) as American Indian or Alaska Native, and 2/20 (10%) as a race not listed. Of the 20 participants, 3 (15%) identified as Latinx, and 2 (10%) patients had limited English proficiency. Most patients (14/20, 70%) had Medicare insurance. All patients had an active patient portal account. One of the interviewed participants reported having used an e-visit before.

**Table 1:** Interview participants baseline characteristics.

Characteristics		Values (N=20)
Age (years), median (IQR)		74 (68.5-77.8)
**Sex, n (%)**		
	Female	7 (35)
	Male	13 (65)
**Race, n (%)**		
	American Indian or Alaska Native	1 (5)
	Asian	5 (25)
	Black or African American	2 (10)
	Other	2 (10)
	White or Caucasian	10 (50)
**Ethnicity, n (%)**		
	Hispanic or Latinx	3 (15)
	Not Hispanic or Latinx	17 (85)
Limited English proficiency		2 (10)
**County of residence, n (%)**		
	San Francisco	14 (70)
	Other	6 (30)
**Insurance, n (%)**		
	Medicare	14 (70)
	Medicare advantage	2 (10)
	Medicaid	3 (15)
	Other	1 (5)

We identified 4 overarching coding categories or themes, comprising 7 codes (Table 2).

These themes stemmed from a combination of both prompted (in response to an interviewer question) and unprompted (ie, spontaneous) comments from participants. First, participants generally were accepting of the concept of e-visits and were willing to try them. For example, after hearing about the effort to make this visit type available to patients, one participant stated “I wholeheartedly endorse that. I think it’s not just a good idea, it’s essential.” Participants voiced that e-visits may be most helpful to prevent office visits. For example, one participant stated, “the actual cost [of an in-person visit] is a lot more because of transportation…and lost opportunity to do something else.” Participants also generally agreed with the idea that providers should be fairly compensated for time spent on medical decision-making, whether in a synchronous visit (eg, video visit) or asynchronous visit (eg, e-visit).

Second, nearly two-thirds (10/16) of the participants voiced a preference for synchronous communication. In many cases, in-person visits were preferred. These preferences stemmed from improved perceptions of communication and comprehension by both patients and physicians.

Third, some participants had specific concerns about e-visits. Many found the name confusing and thought they should instead have a more descriptive name, such as “online medical advice.” Participants also voiced concern about choosing the right visit type in the patient portal and did not think it should be left up to the patient to determine whether they should submit a billable e-visit or nonbillable message. Participants also voiced discomfort expressing medical questions in writing. One participant stated, “You’re relying upon your narrative. You have to express in [the patient portal] within a certain amount of words exactly what the issue is. And believe me, that sometimes can’t be captured.” Only 2 of the 7 participants who expressed this concern had limited English proficiency.

Fourth, 6/18 (33%) participants indicated discomfort using or accessing technology for e-visits. For example, the daughter of one participant stated “[My parents] don’t have the computer skills. They are from the telephone era. They prefer talking to a human being and it makes sense to them versus typing.”

Financial barriers were not a common theme, with only 3/18 (17%) patients expressing concern over increased out of pocket costs resulting from patient portal messages being converted to billable e-visits. Only 3/18 (17%) participants thought that precise out-of-pocket patient costs for e-visits need to be stated up front before a patient sends a message. However, participants did expect that the out-of-pocket costs for e-visits should be less than or equal to the costs for a synchronous visit. The most commonly quoted amount that participants reported they would pay out of pocket for an e-visit was US $20.

There were minimal differences in themes between participants who did or did not use the patient portal tool in the past 6 months. For example, 6/9 (67%) participants recruited from the patient portal group liked the idea of e-visits versus 5/10 (50%) participants recruited from the telephone hotline group. A total of 4 participants recruited from each group reported discomfort using technology.

**Table 2:** Summary of coding categories and codes describing patient perspectives of e-visits.

Higher-order coding category or theme	Code	Proportion^a^, n/N	Sample quotation
Acceptance of the concept of e-visits	Favors the idea of e-visits	11/19	“I wholeheartedly endorse that. I think it’s not just a good idea, it’s essential”
	Willing to try e-visits	14/18	“All of sudden [if I] have sores in my mouth or something like that, I'm sure that could be handled through an e-visit.”
	Thinks e-visits may help prevent office visits	11/16	“The actual cost [of an in-person visit] is a lot more because of transportation…and lost opportunity to do something else”
	Thinks providers should be fairly compensated for their time	11/18	“I am still using his time, so he definitely should get compensated for that because they are giving me medical advice”
Preference for synchronous communication^b^	Preference for synchronous communication^b^	10/16	“[The patient portal] is not very effective because there’s a lot of miscommunication and misunderstanding.” “I’m constantly having to ask for repetition. This is good because it helps me understand if the doctor is understanding me, as well as me understanding the doctor. In a text message you don’t get that.”
Concerns about e-visits	Naming convention is confusing	10/16	“I don’t know what it means…I think you would have to say something like an online visit, or online medical advice”
	Difficulty choosing right visit type within the patient portal	8/14	“the doctor [should] make the call.” “if there were some guidelines, that could help the patient”
	Discomfort expressing medical questions in writing	7/18	“You’re relying upon your narrative. You have to express in [the patient portal] within a certain amount of words exactly what the issue is. And believe me, that sometimes can't be captured.”
Discomfort using technology^b^	Discomfort using technology^b^	6/18	“[My parents] don’t have the computer skills. They are from the telephone era. They prefer talking to a human being and it makes sense to them versus typing…” “I never could remember my password. Well, ok, that’s because I’m old.”

^a^We excluded participants from the denominator if they did not answer the pertinent question(s).

^b^Singular topics that were common within the data set are included as both a code and a theme.

## Discussion

### Principal Findings

The majority of participants in this study were accepting of the idea of e-visits—billable, asynchronous patient-initiated messages—and willing to try one, despite preferring synchronous visits. Most participants agreed that providers should be fairly compensated for medical decision-making. Although they opined that out-of-pocket costs should be similar to or less than the cost for synchronous visits, they did not voice major concerns over additional out-of-pocket spending. Few participants voiced the need to know the precise cost of the visit upfront. Major concerns about e-visits included naming conventions, difficulty choosing the right visit type, and discomfort expressing medical terms in writing. A minority of participants expressed technical barriers to use of e-visits, and these included both participants who had and had not recently used the patient portal to receive care.

Concern about potential out-of-pocket costs was not a common theme among the participants in this study, and participants generally agreed that providers should receive payment for providing medical advice via web-based messages. This finding is important because it differs from the concerns raised in recent media coverage of e-visits, which has emphasized the implementation of billable messaging causing possible financial harm to patients [29,30]. However, this study was limited to mostly patients with Medicare insurance, so the absence of concern about out-of-pocket costs of e-visits may not be generalizable to other patient groups with different health insurance cost sharing structures.

Over the past several years, there has been a major increase in the number of patient portal messages, leading to burnout among frontline nurses, physicians, and other staff who must respond to these messages, largely between or after their other clinical duties and without reimbursement [31,32]. Billable e-visits are one potential solution. By reimbursing providers for delivering medical advice outside of a visit, e-visits may allow them to schedule more protected time during the day for responding to messages, rather than doing so on nights and weekends. They may also encourage care delivery organizations to innovate alternate means of communicating with and engaging patients. However, there is a risk that if organizations replace patient-portal messaging with billable e-visits, it will create a financial disincentive for patients to seek help when they need it. Therefore, the findings of this study are important, as they suggest that the majority of older adults are receptive to the concept of e-visits and do not perceive major financial barriers to use.

Older adults are at particular risk for facing health disparities as a result of decreased access to digital health technologies [33,34]. Our findings include perceptions of older adults and other groups at risk for health disparities, including those with limited English proficiency and of various races and ethnicities. Most participants in this study preferred synchronous communication over asynchronous methods like e-visits and unbilled patient portal messages, putting them at risk for ongoing disparities unless specific efforts are undertaken to make these tools attractive and accessible to them. Various interventions have been suggested for addressing the digital divide in general—for example, universal internet access, training in computer literacy, language concordant materials, and encouraging family or caregiver assistance in using digital tools [34]. In addition, we recommend several interventions below that are specific to e-visits.

**Figure 1:**
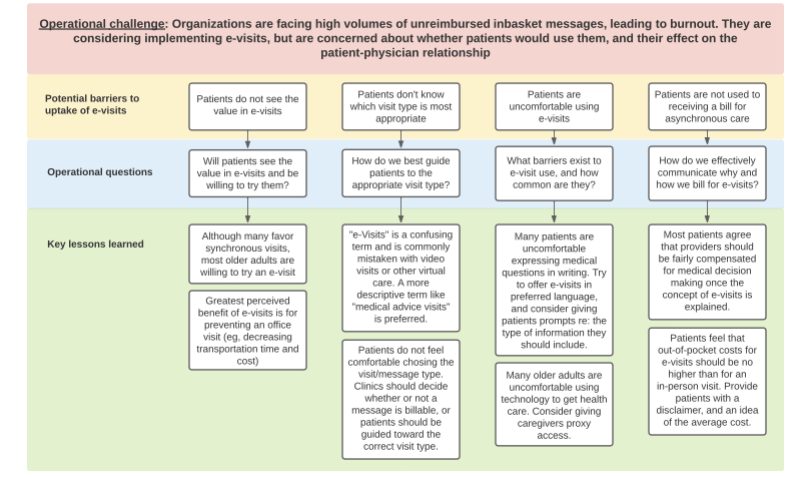
Recommendations for implementation of e-visits in practice.

Given recent changes in Centers for Medicare and Medicaid Services policy that allow for broader use of e-visits, many organizations may be considering implementing e-visits. However, there are several potential barriers to consider. The findings from our analysis, which includes the perspectives of a diverse group of older adults, may be generalizable to other institutions.(Figure 1). We identified several key lessons learned that may inform interventions to improve acceptability and usability of e-visits. First, patients may not know what e-visits are or how to choose between an e-visit and a traditional patient portal message or other visit type. To address this, we recommend that the patient-facing name for e-visits be something more descriptive, such as “medical advice message.” We also recommend that patients not be asked to determine whether their message clinically qualifies as a billable e-visit or a nonbillable patient portal message—rather, that there should be a single point of entry for patients and billing should occur only if the message meets criteria. Third, patients may have technical, language, or educational barriers to using e-visits. We found that few participants actually lacked access to the necessary equipment but that many expressed discomfort using technology or expressing medical questions in writing. For this reason, we recommend that patients be able to write e-visit queries in their preferred language and with prompts (eg, are you having pain? What medications have you tried?) to improve comfort with phrasing medical questions in writing. Patient caregivers (eg, adult children) should be given proxy access to patient portals to submit e-visits on their behalf. Finally, patients may have concerns about receiving a bill for patient portal messages that are classified as e-visits if their patient portal communications had all been unbilled in the past. We recommend that organizations include a disclaimer in plain language that requires the patient’s acknowledgement, describing that some messages may be billed and may generate out-of-pocket costs to the patient. Ideally, organizations should also provide patients with the range or average out-of-pocket cost of these visits, acknowledging that they will vary by insurance type.

Our results align with published literature on patient perceptions of use of other virtual care options, such as patient portals (secure websites giving patients access to personal health information, including the ability to communicate with their care team) and chatbots. For example, a 2018 National Poll by the University of Michigan determined that the technology gaps for older adults were rapidly narrowing, but nonetheless, respondents older than 65 years were more likely to report that they did not like using the computer to communicate about their health [35]. Similarly, a systematic review of perceptions of patient portals [36] determined that patient-provider communication was the most prevalent positive attribute, while concerns over security and user-friendliness were the most prevalent negative perceptions. This sentiment aligns with our findings that participants perceived a benefit of e-visits being the ability to communicate with their physician asynchronously to avoid a visit, while a commonly cited barrier to use was navigating the patient portal to choose the appropriate visit type. This insight also supports the conclusion of a study from Sweden [37] in which patients who used a chat-based, automated history-taking service appreciated the ability to communicate medical information asynchronously and potentially prevent an unnecessary visit.

### Limitations and Strength

This study has several limitations. First, because e-visits were not being frequently used at the time of the study, very few of the participants had used an e-visit before, so their perceptions were based on the description of this new visit type by the interviewer. Patients who have used an e-visit to receive care in the past may have different perceptions. Second, we interviewed a relatively small sample size of 20 patients, and the majority were from the San Francisco Bay Area, where there are high rates of digital literacy. Our findings may therefore not be generalizable to very different patient populations. However, with this study’s sample size and composition, we reached saturation of ideas in participants responses. Third, interpretation of transcription content may be biased due to individual reviewer’s implicit biases. However, neither of the reviewers had a direct role in overseeing e-visit implementation and had no financial or other incentives for e-visits being successful. Fourth, participant responses could have been more favorable due to social desirability bias since the research team is affiliated with the health care system in which they receive care [38]. However, the researchers were not on the patients’ treatment teams and explicitly stated prior to the interview that all responses would be anonymized and would in no way affect their care. Furthermore, gift cards to compensate participants’ time were distributed prior to the interview to prevent any misperception that certain responses would be linked to reward. Fifth, because we used open-ended questions, more time may have been spent discussing certain themes or categories than others, which may have created a bias in quantitative analysis if participant responses were a reflection more so of the questions asked than of their opinions about e-visits. Finally, our study population was identified among UCSF primary care patients who had used a patient portal or telephone triage tool to seek an appointment, testing, or triage advice about COVID-19. It is possible that these patients differed in some way from the general population, though we have no reason to suspect that this group would have different perceptions about virtual care.

The study also had several strengths. We recruited a group of participants with diverse personal characteristics, including race, ethnicity, language, income, and geography, to capture of range of experiences. We allotted up to 60 minutes for each interview to provide time for open responses and for participants to elaborate on their responses. We also recruited patients who had variable historic use of the patient portal, to capture the perceptions of those who may be less technically savvy with digital health tools.

### Conclusion

In summary, this is one of the first studies to report qualitative feedback on e-visits, and the first, to our knowledge, to do so since the start of the COVID-19 pandemic and reflecting the 2020 changes Centers for Medicare and Medicaid Services policy changes related to e-visits. Our evaluation is an important step toward understanding patient perceptions around e-visits, a relatively new asynchronous form of digital health care delivery. Our findings suggest that older adults are generally accepting of the concept of e-visits, but still prefer synchronous visit types. We identified opportunities to improve e-visit implementation and design. As telemedicine and virtual care continue to grow and occupy a greater part of the health care landscape, issues such as patient acceptance, digital health care access, and usability in provider workflows will become increasingly critical to the success of such programs.

## Appendix

Multimedia Appendix 1 Structured interview guide.

## Abbreviations

UCSF: University of California, San Francisco
