# Supplementary material for: Patient Perceptions of e-Visits: Qualitative Study of Older Adults to Inform Health System Implementation
Source: JMIR Aging. 2023 May 26;6:e45641. doi: 10.2196/45641 (PMC10257108; doi:10.2196/45641)
Supplement: Multimedia Appendix 1 [file aging_v6i1e45641_app1.docx]

**Appendix 1: Interview script**

**Introduction**

Thank you for agreeing to speak with us today. We are researchers at UCSF who are working on
ways to better connect patients to the healthcare they need without having to come into the
doctor’s office. This type of health care is called remote care, and we hope that these remote
care options will save patients time, improve communication, and allow them to receive
excellent care.

Today we're going to be talking about two topics: the ways you currently communicate with
your healthcare team, and your thoughts about a type of message called an E-visit. An E-visit is
a message to your doctor asking for medical advice. The idea is that these messages can be
used to get medical advice from your doctor, without having to take the time to go into the
office, or have a video visit. We’ll give you some more information about E-visits later in the
call.

We are interested in hearing your personal perspective. There are no wrong answers;
everything you say is valid and true. Also, if we ask anything that feels too personal, you can
simply ask us to move to the next question. You don't have to answer anything you don't want
to.

Because it’s difficult to recall our entire conversation, and so that we can focus on you, we'd
like to record and take notes during this session. We will not be saving any identifiable or
personal information with the recording or notes. We will transcribe the recording and
everything will be anonymized. Are you ok with us recording and taking notes?

You will be getting a $60 gift card for your participation. It is already being sent/ delivered to
you. It is a way to thank you for your time and ideas.

Any questions before we begin?

**Guiding questions**

1. I’d like to begin with learning about how you typically communicate with your providers.
   What methods do you use/have you ever used to get help when you have a medical question or need medical advice from your doctor?
   1. What has been your experience with sending messages or asking questions via
      MyChart? How has it been helpful in communicating with your doctor(s)??
   2. When would you not want to communicate with your doctor through MyChart?
   3. What other types of things do you use MyChart for? (e.g. refilling your
      medication, asking new medical questions, arranging your next appointment,
      viewing after visit summaries, labs)
   4. If MyChart is not mentioned: Did you know that UCSF has a patient portal called MyChart for viewing health information and communicating with your providers? What are some reasons that you haven’t used MyChart?
2. Now we’d like to get your thoughts about the different types of messages in MyChart.
   MyChart messages are a popular way for patients to communicate with their doctors and healthcare team. These are good for simple questions like “where is the lab, and when should I go for my blood draw?” These will never cost the patient anything, and insurance companies will not get a bill. That’s ok for doctors, since these messages can usually be handled by nurses or medical assistants, and usually take only a minute or two to respond to. However, when patients send MyChart messages that ask for medical advice, it may take doctors a long time to respond with the right advice for that patient. If
   doctors are responding to dozens of these messages every day, they may have less time to see their patients, and often stay up late at night responding to them. E-visits are a different type of message in which patients ask for medical advice. They give doctors credit for providing written medical advice. For example, let’s say you send your doctor a picture of a new rash, describe how the rash feels, and ask the doctor what they think it is, and how it can be treated. The doctor will take time to read your question, look through your chart, and then offer medical advice and a plan for diagnosis or treatment. Insurance companies will be billed for these e-visits, and that means that you may get a bill for the copay. The major advantage of this visit to you is that it may save you from having to go in for an in-person visit, or take time out for a video visit. Is there anything that I’ve just described about an e-visit that is unclear or confusing?
   Based on what I just explained about e-visits:
   1. Have you ever used them?
   2. What are your initial feelings about eVisits?
   3. Would you consider using them if you needed medical advice? If yes, why? If not, why not?
   4. Which of your doctors, if any, would you feel comfortable doing an e-visit with?
   5. Let’s say you need medical advice from your doctor. When deciding whether to do an e-visit or request an appointment, how important would the following things be for using an e-visit?
      1. You have known your doctor for a long time
      2. You have complete trust your doctor
      3. You have done other types of virtual care with this doctor before (like
         messages, phone calls or video visits)
      4. The type of health problem you have (e.g. that you think it’s well-suited
         for an e-visit)
   6. What do you think about the name “eVisit.” Do you think it makes sense?
   7. How would you describe this type of visit to a family member or friend?
3. Because E-visits involve medical advice from your doctor and may require a significant amount of their time, they are billed to your insurance. This may result in a copay or co-insurance that the patient is responsible for paying. What amount would you be willing to pay as a co-pay for an E-visit?
   1. If it is not possible to know up front the exact amount of the co-pay, would you still do an E- visit?
4. Next, we want to get your thoughts about how to make e-visits useful when you need to medical advice from your doctor. First, which of the following would be the best way to help you in choosing whether a question should be sent as an e-visit or a MyChart message?
   1. Let you decide the whether the question should be sent via an e-visit or sent as a regular message.
   2. You send one type of message, regardless of whether it is asking for medical advice or not – and the doctor or their staff decides whether it’s a billable e-visit or a regular MyChart message.
   3. You answer a series of questions in the patient portal that guides you to the
      right choice
5. Which of these messages about billable e-visits would most convince you to use them?
   1. Benefits to you, like being able to get medical advice without playing phone tag, or taking time off for a visit
   2. Examples of situations to help you understand when to use an e-visit (e.g. a new rash)
   3. The reason that these visits are helpful to your doctors (that doctors get reimbursed for providing online medical advice
6. Some of the common reasons for an e-visit at other health systems are listed below. Would you use an e-visit for any of these?
   1. A new symptom like a rash or symptoms of a urinary tract infection
   2. Worsening of a chronic condition you have (like uncontrolled high blood pressure)
   3. A question about a medication you’re taking
   4. A question about a test result
